# Momentum Distribution of Near-Zero-Energy Photoelectrons in the Strong-Field Tunneling Ionization in the Long Wavelength Limit

**DOI:** 10.1038/srep11473

**Published:** 2015-06-17

**Authors:** Q. Z. Xia, D. F. Ye, L. B. Fu, X. Y. Han, J. Liu

**Affiliations:** 1 National Laboratory of Science and Technology on Computational Physics, Institute of Applied Physics and Computational Mathematics, Beijing 100088, China; 2 CAPT, HEDPS, and IFSA Collaborative Innovation Center of MoE, Peking University, Beijing 100871, China

## Abstract

We investigate the ionization dynamics of Argon atoms irradiated by an ultrashort intense laser of a wavelength up to 3100 nm, addressing the momentum distribution of the photoelectrons with near-zero-energy. We find a surprising accumulation in the momentum distribution corresponding to meV energy and a “V”-like structure at the slightly larger transverse momenta. Semiclassical simulations indicate the crucial role of the Coulomb attraction between the escaping electron and the remaining ion at an extremely large distance. Tracing back classical trajectories, we find the tunneling electrons born in a certain window of the field phase and transverse velocity are responsible for the striking accumulation. Our theoretical results are consistent with recent meV-resolved high-precision measurements.

The above-threshold ionization (ATI) phenomenon of atoms exposed to a strong field has attracted sustaining attention for decades since it was first discovered in 1979^1^. One of the most pronounced features of ATI in the long-wavelength limit is the high-energy photoelectron spectrum plateau extending up to 10*U*_*P*_ (*U*_*P*_ = *I*/4*ω*^2^, denotes the ponderomotive energy, where *I* is the laser intensity and *ω* the frequency in atomic units)^2^,^3^. The underlying mechanism has been attributed to the tunneled electron’s multiple returns and rescattering by its parent ion^4^,^5^,^6^,^7^,^8^,^9^,^10^,^11^.

Recently, some unexpected low-energy structures (LES) of ATI^12^,^13^,^14^ were observed in the tunneling regime, initially at several eV and then at lower energies of less than 1 eV, triggering a new surge of attention for ATI. Although from many aspects, including quantum and semiclassical, numerous theoretical investigations^12^,^13^,^14^,^15^,^16^,^17^,^18^,^19^,^20^,^21^ of the underlying physics of LES were reported, the controversies on the surprising structure have been continuing. In the above experiments, the photoelectrons were detected only within a small solid angle around the laser polarization direction by a time-of-flight spectrometer. Because LES is subtle and sensitive, it is difficult to disentangle the origin of the various findings without large solid angle measurements including momentum information with high resolution. Recent experimental work by J. Dura *et al.*^22^ stepped forward in this direction. In the experiment, with a specifically developed ultrafast mid-IR light source of 3100 nm in the combination with a 3D reaction microscope, the strong-field dynamics was explored in three-dimensional momentum space down to meV energies with an unprecedented precision^22^. Instead of structures on the eV level, an apparent meV electron accumulation in the ATI spectrum and a striking momentum distribution for the near-zero-energy electron were observed. Nevertheless, the physics underlying the meV electron distribution has not been settled and urgently calls for a theoretical investigation.

In this paper, stimulated by the recent experiment and attempting to resolve the controversies on LES, we theoretically investigate the ionization dynamics of Argon atoms in intense laser fields within the deep tunneling regime of 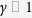 (Keldysh parameter 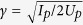, where *I*_*p*_ is the ionization potential), with special emphasis on addressing the momentum distribution of the near-zero-energy photoelectrons. Our study is facilitated by an improved semiclassical rescattering model that includes the Coulomb attraction and trajectory interference and can precisely produce the momenta of the near-zero-energy photoelectrons regardless of the Coulomb long-range tail. Our theory accounts for the surprising accumulation around near-zero momenta corresponding to meV energies and predicts a “ V”-like structure at slightly larger transverse momenta. We identify the roles of the Coulomb attraction and trajectory interference, by tracing back the trajectories of soft and chaotic scattering, respectively. Our work provides profound insight into the meV low-energy ATI mechanism, and helps to unravel the debate about LES in combination with the recent high-precision experimental results.

## Results

We have made simulations for Ar atoms with *I*_*p*_ = 0.583 a.u.. The laser parameters are chosen as *ε*_0_ = 0.053 a.u. and *ε*_0_ = 0.0147 a.u. (λ = 3100 nm) to match the experiment^22^. Thus, the Keldysh parameter *γ* = 0.3. The laser pulse envelope is half-trapezoidal, constant for the first six cycles and ramped off linearly within the last six cycles as in equation (1) in Method section. After the laser pulse, the instantaneous positions and momenta of the emitted electrons are recorded. However, the instantaneous momenta are not equal to the asymptotic ones (i.e., *r* → ∞) collected by the detector due to the Coulomb long-range tail. To precisely reproduce the near-zero momentum distribution, we need to extract the asymptotic momenta from the instantaneous positions and momenta. The Coulomb two-body system has three conserved quantities: the energy *E*, the angular momentum 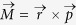 and the Laplace-Runge-Lenz vector 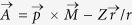. Using these conserved quantities, we can then obtain the asymptotic momenta of the emitted electrons as 
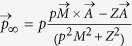
, where Z is the charge and 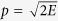
23. Besides the semiclassical simulation, a simple model which assigns a phase to each trajectory is used to address the quantum interference effect. The details of the simulations are presented in Method section.

### Momentum Distribution of Low-Energy Electrons

**Figure 1:**
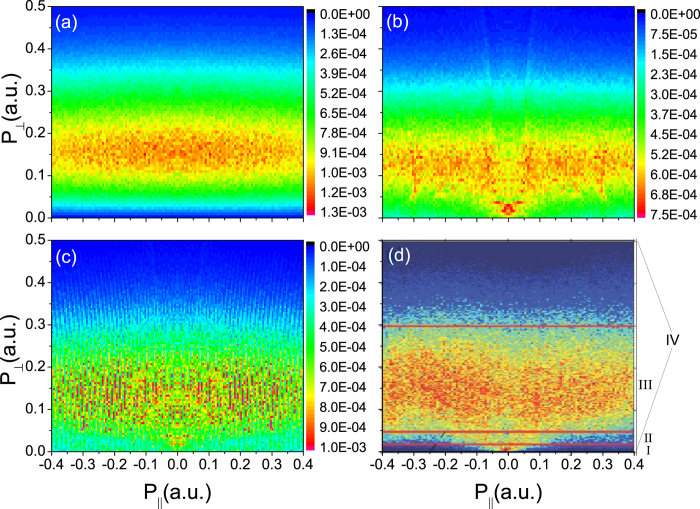
(**a**) Momentum distribution calculated from the model without considering the Coulomb attraction in rescattering; (**b**) Results of the model with considering the Coulomb attraction; (**c**) Results of the model considering both Coulomb field and trajectory interference; (**d**) Momentum spectrum from experiment cited from the reference^22^ for comparison.

Figure 1(a–c) show our model calculations on the momentum distribution spectra of low-energy electrons in the momentum ranges 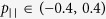 (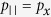, i.e., the momentum along the laser polarization direction) and   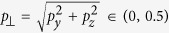, indicating the prominent roles of both the Coulomb potential and trajectory interference.

We can first see the important role of the Coulomb attraction by comparing Fig. 1(a) with (**b**). In Fig. 1(a), we artificially remove the Coulomb potential in the post-tunneling scattering. Here, the momentum spectrum exhibits a simple “sandwich”-like structure, with the dense distribution in the central belt only reflecting the Gaussian type distribution of initial transverse velocities. In this case, we can not see any accumulation near zero momentum. However, in the presence of the Coulomb potential (see Fig. 1(b)), we can obviously observe the accumulation near zero momentum as well as a “V”-like structure at the slightly larger transverse momenta.

The above classical trajectory evolution, however, ignores the quantum interference totally. Comparing Fig. 1(c) with (**b**), we find that the role of the trajectory interference becomes obvious: the dense belt structure broadens and the accumulation near origin fades out a little, leading to better agreement with the experiment. Figure 1(c) also shows the fine vertical interference contrasts. These interference patterns originate from the orbits released in a single cycle with the same final momentum^24^, i.e., the so called intra-cycle interference^25^. It can be expected that there is no such interference but ATI pattern with elliptically polarized laser fields^26^, since in that case the final momenta of these orbits from the same optical cycle will be different along the minor axis.

**Figure 2:**
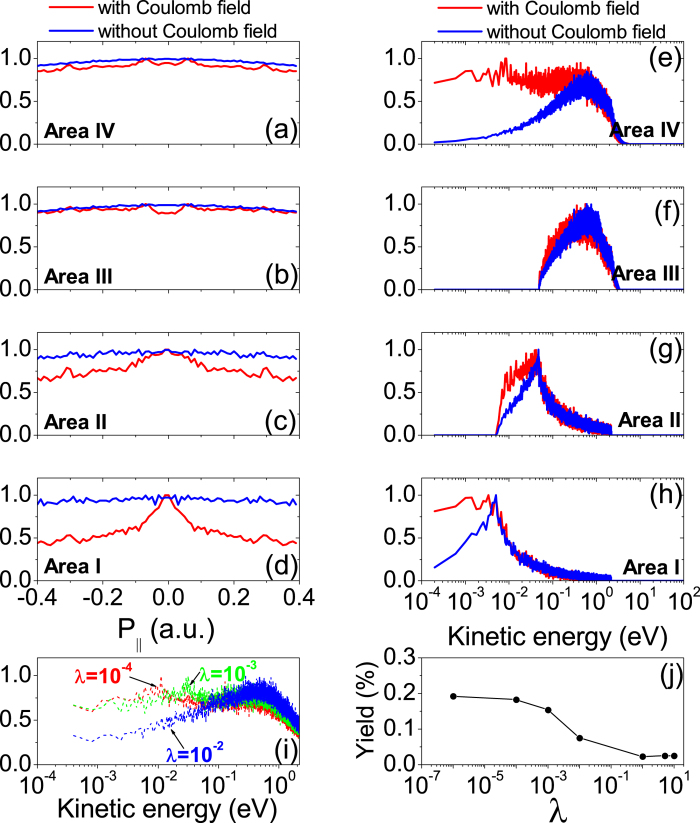
(**a**–**h**)The parallel momentum distribution and the energy distribution obtained from Fig. 1(b), with respect to the four areas within the momentum map of Fig.1(a,d and e) correspond to the area IV; (**b**) and (**f**) correspond to the area III; (**c**) and (**g**) to the area II; and (**d**) and (**h**) to the area I. Red curves denote the results of the semiclassical simulation with the Coulomb field, while the blue ones represent the simulation results without the Coulomb field. (**i**) and (**j**) are simulation results from screening potentials: (**i**) Low energy distribution with respect to screening parameters; (**j**) meV photoelectron yields vs. the screening parameters.

To achieve deeper insight into the origin of the striking structure in the momentum distribution and address its relation to the energy spectrum, we perform statistics and generate energy distributions with respect to distinct transverse momentum regimes, i.e., accumulation regime (I), V-structure regime (II), and belt regime (III). Symbol IV represents the sum of the above three regimes. In our energy statistics, the energy interval is chosen as 0.4 meV, consistent with the experimental resolution. The results are shown in Fig. 2.

From Fig. 2(f–h), we see the Coulomb effects increase and become very significant at small transverse velocities. The envelope of the total energy spectrum (Fig. 2e) shows a prominent hump around 0.5 eV and then extends to meV energies or even less. The hump is also apparent in experiment^22^. Nevertheless, it has nothing to do with the Coulomb attraction, but is just due to the momentum space density compression when the momentum distribution is transformed to energy distribution. An estimation of the hump center is given by 
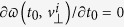
 and 
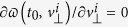
, where *ϖ* is the ionization probability via ADK formula. In the long-wavelength limit of *ω* → 0, we analytically obtain an electron kinetic energy of 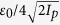, where *ε*_0_ is the maximum field strength. Substituting the atom and laser parameters, it gives 0.33 eV, in close agreement with the results of the simulation. Besides that, the total energy distribution also displays the signature for the slow electron accumulation around 8 meV, which is consistent with the peak near 1 meV observed in the experiment^22^ within the energy resolution of 11 meV. But in contrast to the experiment, below the accumulation, our simulation exhibits a plateau structure in energy spectrum that spreads down the regime of 10^−4^ eV or less.

The meV electrons are closely related to the Coulomb attraction. In particular, we find that the long-range Coulomb attraction between the electron and ion plays a crucial role, in contrast to the Coulomb focusing effect^27^ that is significant only when the electron is closer to ion. We have replaced the Coulomb potential by a Yukawa type potential of −*exp*[−λ*r*]/*r*, to screen the Coulomb long-range tail (see Fig. 2(i,j)). We find surprisingly that, even with a very small screening parameter of λ = 0.01, the meV electron yield decreases rapidly, analogous to the case where the Coulomb potential is completely absent. Only with much smaller screening parameter of 0.001 or less, can the meV electron accumulation be recovered. The above observation unambiguously indicates the crucial role of the Coulomb attraction between the escaping electron and ion at the extremely long distance (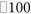 a.u.), and provides the strong evidence that the highly excited Rydberg states are involved in the meV electron dynamics. We have also calculated the meV electron yields (i. e. energy less than 0.01 eV) with respect to the screening parameters in Fig. 2(j), which mainly exhibits a logarithm feature. The singularity stemming from the Coulomb long tail also emerges in heavy ion impact ionization, manifesting a sharp cusp-like peak at zero transverse momentum^28^,^29^. Recently, the cutoff of Coulomb potential at the distance of a few atomic units is found to affect the momentum spectra of the electrons with eV energy in multiphoton regime^30^,^31^, and some evidences have been presented that the low energy structure at several eV originates from the long-range Coulomb interaction^32^. Here, we find that the tiny Coulomb tail at a distance much larger than 100 a.u. can significantly help produce the meV photoelectrons and lead to the striking distribution in momentum spectrum.

In addition, we find that the surprising accumulation around near-zero momenta is laser wavelength dependent. When we decrease the wavelength to 800 nm (i.e., larger field frequency), the apparent accumulation is no longer observed. The phenomenon can be attributed to the high-excited Rydberg states which are deeply involved in the meV photoelectron generation. When the field frequency is larger than the frequency of the classical orbit of the Rydberg state, the electrons pumped into the Rydberg states become stabilized against ionization^33^,^34^. This effect can reduce the meV electron accumulation. And it may be also related with the lack of Coulomb correction of photoelectrons for low ellipticity light at 800 nm^35^ which is explained by the recapture process^36^. Since in the simulation it is demonstrated that at 3100 nm some Rydberg electrons may relax to continuum states during the switching off of laser pulse, the Coulomb effect could display some special signature in the long-wavelength limit.

### Classical Trajectory Analysis of the Source of the meV Electrons

**Figure 3:**
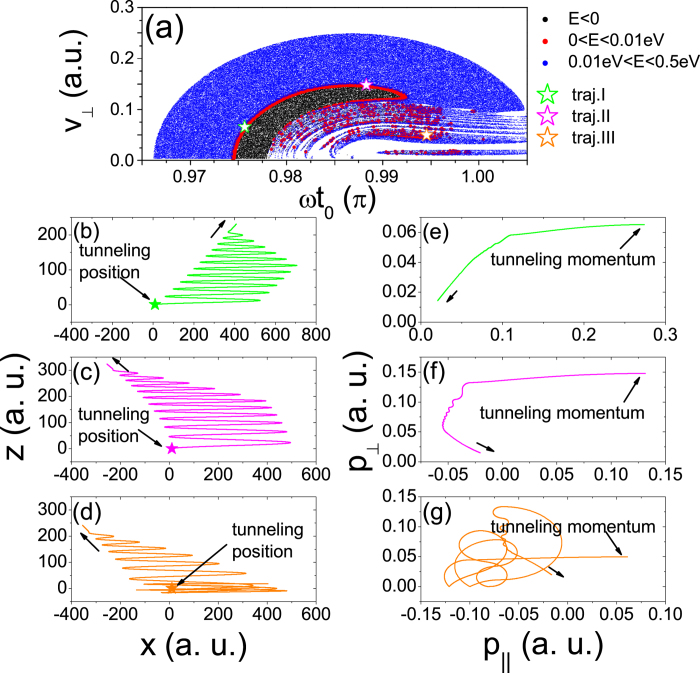
(**a**) The dependence of final kinetic energy on tunneling phase and initial transverse velocity around peak field. The blank areas contribute to high-energy electrons with *E* > 0.5 eV. Three typical trajectories (**b**–**d**) leading to meV energy and their corresponding temporal evolutions of the momentum (**e**–**g**). (**b**) and (**e**), the soft forward scattering trajectory; (**c**) and (**f**), the soft backward scattering; (**d**) and (**g**), the chaotic scattering.

In our semiclassical model, the emitted electron’s energy is determined by the tunneling phase and initial transverse velocity. In Fig. 3, we show the dependence of the final kinetic energy on the tunneling phase and initial transverse velocity around peak field. The meV electrons originate from the red area which consists of a regular arc region and scattered irregular regions below the arc. The irregular zone is self-similar and has fractal properties^10^,^37^,^38^,^39^. The trajectories originating from this region might experience multiple returns to the ion and the final energies are very sensitive to the initial conditions. The chaotic multiple rescatterings by the Coulomb field might lead to extremely high energy electrons that are responsible for the well-known ATI plateau structure^10^. It can also result in extremely low-energy electrons, as shown by the scattered red dots below the arc region.

Besides these chaotic trajectories, we find the electrons with meV energy usually experience soft scattering and then move forward or backward^16^,^21^. We plot such three kinds of typical trajectories in Fig. 3(b–d), respectively. Their initial conditions correspond to the “ stars” in Fig. 3(a) . Temporal evolution of the momentum for each trajectory is shown in Fig. 3(e–g), separately. Since the electrons oscillate in the laser field, here we use the canonical momentum^41^ 
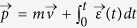
 instead of the mechanical momentum where 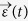 represents the electric field. Fig. 3(b,c,e and f) indicate that some electrons originating in a certain window of laser phase and initial transverse velocity can tunnel into Rydberg states without ionization. These electrons are located far away from the parent ion and are weakly bounded by the ion’s Coulomb attraction. They subsequently experience very soft rescattering during which they can only acquire limited field energy to be pumped into the continuum with meV energy. During the process, the Coulomb potential attracts the electron and reduces the electron’s tunneling momentum to zero showing a kind of “friction” effect (see Fig. 3(e,f)). While for the chaotic trajectory, the electrons experience multiple scatterings with ion and are “ occasionally” emitted with meV energy. Our model calculation indicates that both chaotic and soft (forward or backward) recattering trajectories are the source of meV photoelectrons. Since the final atomic energy varies rapidly with the tunneling phase and initial velocity during chaotic scattering, we estimate that the ratio of the two kinds of events (i.e., chaotic events vs. soft rescattering events) is no more than 1/4. The above observation is similar to those for Rydberg atoms^34^,^41^.

## Discussion

Since 2008, strong-field ionization with low-frequency field has revealed the unexpected low energy structures of ATI. In order to address the topic, recently the experiment by J. Dura *et al.*^22^ provided high-resolution 3D electron momentum data for strong-field ionization in the long wavelength laser field, and observed surprising new electron dynamics of near-zero-momentum electrons and extremely low momentum structures. The current calculations in the paper obtain a good agreement with the experimental results. However, we observe that the accumulation near origin in the momentum spectrum may be blurred if the pulse envelope is changed in the simulation. For example, if the electric field is switched off in a cos-squared form instead of the linear ramping off as listed in the Method section, the zero-momentum accumulation will be strongly inhibited. On the other hand, if only the duration of the linear switching-off is changed, e.g., spending 5 or 7 cycles to turn off instead of 6 cycles, the accumulation and the “V”-like pattern as well as other features of the momentum distribution can be maintained qualitatively, but the position of accumulation will shift along the 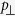 axis. The effect of the shape of pulse envelope has been reported by I. A. Ivanov *et al.*^43^ and is named as “ displacement effect” there. More simulation results about the effect of pulse envelope are presented in the Supplementary Material. These observations demonstrate that the generation of the near-zero-energy electrons is very sensitive to the pulse envelope, which may be related to the dynamical stabilization of atoms, and more deep experimental and theoretical study are needed.

**Figure 4:**
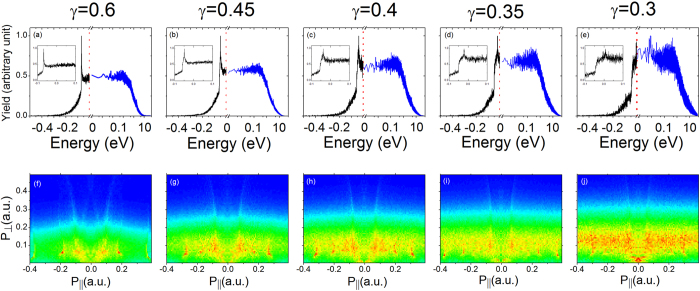
Electron energy distributions (upper panel) and photoelectron momentum distributions (lower panel) at wavelength λ = 3100 nm for varied Keldysh parameters. In the upper panel, black curves denote the Rydberg state distributions and blue curves denote the photoelectron energy distributions. Notice that the Rydberg state distributions are plotted on linear scale while the photoelectron energy distributions are plotted on log scale. The energy distributions on linear scale around zero are plotted in the insets.

Furthermore, in order to investigate the relation between the feature in momentum spectrum and the characteristics of energy distribution more directly, we make simulation for Ar atom under different laser parameters. In Fig. 4, the laser intensity is varied while the wavelength is fixed at 3100 nm. In the upper panel of Fig. 4, we show the distributions of both Rydberg states (negative energy) and photoelectrons (positive energy). Notice that the Rydberg state distributions are plotted on linear scale while the photoelectron energy distributions are plotted on log scale. In Fig. 4(a–d), we can observe prominent peaks in the Rydberg state distribution, and the peaks are found to shift to the right and just cross zero energy at *γ* = 0.3 as shown in Fig. 4(e). Accordingly, in the lower panel, we observe that the narrow accumulation emerges at the origin of the momentum distribution only in Fig. 4(j) corresponding to *γ* = 0.3. We have verified the relation between the momentum spectrum and energy distribution by changing the laser wavelength in simulation, and always observe similar transition in the momentum spectrum when the energy of Rydberg state approaches 0. However, because of the sensitive dependence of the distribution of quasi-bound electron on the laser pulse envelope as we discussed above and in the Supplementary Material, the prominent Rydberg electron peak as well as the accumulation near zero momentum may not been observed if the laser envelope is changed. But the calculation presented in the current paper proposes that when the laser field is turned off, Rydberg electrons should relax to high-lying bound states^42^, even if to meV-energy continuum states. The latter process may induce the slow electron accumulation. More accurate quantum calculations are in demand to clarify the detailed process.

In summary, we have investigated the dynamics of meV ATI photoelectrons from theoretical side for the first time. Our simulations indicate that the meV electron generation is subtle and attributed to the extremely long-range Coulomb tail, while it is also of universality in the deep tunneling regime. Our theoretical results account for the recent high-precision ATI experiments. The predicted photoelectron spectrum can spread down to the energy of 10^−4^ eV or less, and the wavelength-dependence of the electron accumulation calls for further experimental verification. Moreover, besides the calculations on displacement effect in the work of I. A. Ivanov *et al.*^43^, the dependence of the near-zero-energy electron on the laser pulse needs a more detailed scrutiny including full quantum effects. The intuitive model depicted in the current research has addressed the quantum interference effect, but many progresses are expected to make along the routine, such as the improvement of the weight and phase of each trajectory.

In the semiclassical model, the atomic ionization consists of two essential physical processes, i.e., an electron tunnels through the Coulomb field that has been dramatically suppressed by the laser field, and the released electron is driven by laser field to scatter with its parent ion^10^. According to Landau’s effective potential theory, Schrödinger’s equation written in parabolic coordinates (*ξ*,*η*,*ϕ*) can be separated into two one-dimensional equations, and there is a potential barrier along *η* direction. Suppose that the electron tunnels only along the direction of electric field, the coordinate of the tunnel exit, i.e., *η*_0_, can be calculated^10^ from 
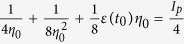
. The tunneled electrons at the tunneling exit have initially zero longitudinal velocity and a Gaussian transverse velocity distribution. Each trajectory is weighted by the ADK ionization rate 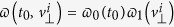
44,45,46, where 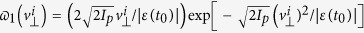 is the distribution of initial transverse velocity, and 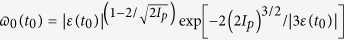, depending on the field phase *ωt*_0_ at the instant of tunneling as well as on the ionization potential *I*_*p*_. In the post-tunneling process, the electron evolution in the combined oscillating laser field and Coulomb field is traced via the classical Newtonian equation 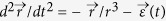. After the laser field is switched off, we calculate the asymptotic momentum as we mentioned above to simulate the time-of-flight process. After that, the physical quantities can be calculated through weighted averaging over the ensemble of trajectories corresponding to diverse initial laser phases and transverse velocities at tunneling. In our simulation, more than 5 million trajectories are calculated and the convergence of the results has been tested by increasing the number of trajectories.

Notice that, as we discussed above, the simulation result is sensitive to the pulse envelope. In the calculation presented here, we are restricted to only considering the electrons released within the first cycle 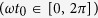. The electric field is used as 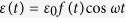, where the half-trapezoidal pulse envelope *f*(*t*) used in simulation is set as equation (1)


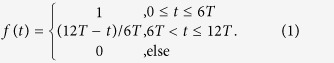


To retrieve the interference effect, for each trajectory we assign a phase *S* by the integral 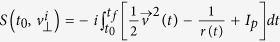
21, where 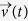 and *r*(*t*) are the solutions of the Newton equation with the initial condition *t*_0_, 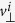 and tunneling position *r*_0_. Then, the transition amplitude from the initial state to the continuum state with the asymptotic momentum 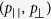 can be calculated as 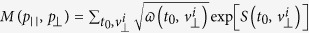, where the summation includes all the trajectories processing the same final momentum 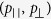. The momentum spectra can be obtained from 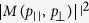.

## Additional Information

**How to cite this article**: Xia, Q.Z. *et al.* Momentum Distribution of Near-Zero-Energy Photoelectrons in the Strong-Field Tunneling Ionization in the Long Wavelength Limit. *Sci. Rep.* **5**, 11473; doi: 10.1038/srep11473 (2015).

## Supplementary Material

Supplementary Information
