# Supplementary material for: Momentum Distribution of Near-Zero-Energy Photoelectrons in the Strong-Field Tunneling Ionization in the Long Wavelength Limit
Source: Sci Rep. 2015 Jun 17;5:11473. doi: 10.1038/srep11473 (PMC4469979; doi:10.1038/srep11473)
Supplement: Supplementary Information [file srep11473-s1.pdf]

Supplementary Information

To manuscript titled:

"Momentum Distribution of Near-Zero-Energy Photoelectrons in the Strong-Field Tunneling Ionization in the Long Wavelength Limit"

By authors: Q. Z. Xia, D. F. Ye, L. B. Fu, X. Y. Han, and J. Liu

In the Supplementary material, we present some numerical results with the pulse profiles different from half-trapezoidal one as Eqs. (1).

1 Effect of pulse envelope

In the simulation, it is observed that the energy of electrons in concern is so small that it is sensitive to the shape of pulse envelope. The momentum distribution and energy distribution are plotted in Fig. S1 (a) and (b) when the pulse is switched off in a cos-squared style instead of linear style, i.e., the pulse profile $f(t)$ is used

as equation (s1) instead of equation (1) in the paper,

$$f(t) = \begin{cases} 1 & ,0 \le t \le 6T \\ \sin^2(\frac{(12T-t)}{6T}\frac{\pi}{2}) & ,6T \le t \le 12T \\ 0 & ,\text{else} \end{cases} \qquad (s1)$$

In that case, the momentum distribution near the origin is in sharp contrast with that in Fig.1 (b). The only different parameter is the style turning off the pulse. In order to address the difference, we calculate the motion of the electron released at $t = 0$ with initial velocity $\vec{v}_0 = 0$ by $v(t) = -\varepsilon_0 \int_0^t f(t) \cos \omega t dt$, and $x(t) = \int_0^t v(t)dt$. While in both cases the final velocity $v(t_f) = 0$, the final position is quite different as demonstrated in Fig. S1 (g). The electron stays around $-\frac{\varepsilon_0}{\omega^2}$ after the pulse in equation (s1), but $x(t_f) = -\frac{2\varepsilon_0}{\omega^2}$ after half-trapezoidal pulse in equation (1). The Coulomb effect is neglected here. With the Coulomb potential taken back into account, the difference in final position implies that the electric field with half-trapezoidal envelope drags some electrons far away from the ion and could transfer energy to them during the pulse switching-off. The effect can be confirmed by the energy distribution in Fig.S1 (b). The onset of the Rydberg electron is around $-\frac{\omega^2}{\varepsilon_0}$ after the cos-squared style pulse, but for the half-trapezoidal one it is obviously shifted to higher energy.

It is interesting that the so-called "displacement effect" of laser pulse shape has already been reported very recently by I. A. Ivanov et al.[1]. In their work, by changing the ramp on/off profile of laser pulse from linear to cos-squared similarly as we did here, they demonstrated that this seemingly insignificant change could have a dramatic effect on the photoelectron spectrum and the photoelectron angular distribution. Though they worked on the XUV pulse parameters there, they claimed that this effect appears to be general. Since their results are based upon high accuracy quantum calculation, their work confirms our observation using classical trajectories approach. Based upon the calculations here, it can be supposed that the displacement effect of laser pulse envelope may deeply influence the energy of the Rydberg electrons and the meV electrons as well as the accumulation of electrons near the origin, and the "displacement effect" may depend on the experimental parameters, such as laser intensity, frequency as well as the Keldysh parameter. This effect can help to understand why the simulation in the paper is sensitive to the shape of the pulse envelope. Neither the envelope in equation (1) nor in equation (s1) contradicts with any physical law as mentioned by I. A. Ivanov et al.; hence it needs further theoretical and experimental research

to determine which kind of pulse is more suited for the realistic scenarios.

2 Symmetric pulse

In this part, we present numerical results with symmetric pulse envelopes. Different from the implementation in the paper, here the electrons are released at every moment of the whole duration of the laser pulse instead of only during a single cycle. Two kinds of pulse profiles are used. The first is the full-trapezoidal envelope, i.e., 6-cycle switching up, 6-cycle flat top, and 6-cycle switching off. In Fig. S1 (c), it can be observed that, not only the accumulation near zero but also other low-energy structures near $p_{\|} = 0.2$ and $p_{\|} = 0.3$ are blurred. It suggests that the electrons released at the same tunneling phase but different cycles could acquire different energy and different momentum from the laser field.

Second, since in realistic few-femtosecond laser pulse, only the cycle near the peak contributes to the majority of the ionization events, we use a hybrid bell-

shape envelope as that:

$$f(t) = \begin{cases} \frac{3}{4}\cos^2(\frac{|t|}{4T}\frac{\pi}{2}) + \frac{1}{4} & ,|t| \leq 4T \\ \frac{1}{4}\frac{(9T-|t|)}{5T} & ,|t| \leq 9T \\ 0 & ,\text{else} \end{cases} \qquad (s2)$$

The motivation with which we use the hybrid bell-shape envelope has been discussed above, i.e., the generation of low-lying photoelectrons is benefit from the position shift of electron during the switching-off of pulse. As a consequence, the profile in Eqs. (s2) is a hybrid of cos-squared and linear. The temporal profile of electric field is plotted in Fig. S1 (h), and the evolution of a free electron released at $t = 0$ in that pulse is illustrated in Fig. S1 (g) with black curve. The position shift from the average position $-\frac{\varepsilon_0}{\omega^2}$ is demonstrated while the final velocity remains 0. Identical with the envelopes in equation (1) or (s1), this hybrid envelope in equation (s2) does not contradict with Maxwell's equations either. The final momentum map plotted in Fig. S1 (e) evidently demonstrates that some electrons accumulate near $p_\perp = 0.02$ similarly as those presented in Fig. 1, but with a more realistic and symmetric pulse profile and all the electrons taken into account. Though this accumulation structure is so slender compared with Fig.

1 (b) that the corresponding accumulation feature is not obvious in the energy distribution in Fig. S1 (f), it is still demonstrated that the Rydberg state distribution shifts to higher energy compared with the red curve in Fig. S1 (b). The numerical results with the two kinds of symmetric envelope confirm the relation between the Rydberg state electrons and the meV photoelectrons.

As a conclusion, the implementation of the semi-classical method which we used in the work is standard and has been proven by successfully explaining various strong-field ionization processes. Not only the half-trapezoidal pulse envelope but also the first-cycle release of the tunneling electron have been applied to study many topics about the ionization in strong field [2], especially the related low-energy structure[3,4]. Furthermore, because of the exponential dependence of the ionization rate on the electric field, the majority of ionization events are estimated to be promoted by the cycle around the peak, especially in the short-pulse laser experiment. In order to grasp this feature, and at the same time for the simplicity, we only consider the electrons released within the first cycle of this half-trapezoidal pulse as the implementation of the work. The major part of the simulation results can be reproduced even with the more realistic conditions taken into account. The effect

of the shape of pulse envelope can indeed influence electrons near zero energy as presented here. Following the work by I. A. Ivanov et al.[1], treating the ionization process within the Kramers-Henneberger picture may help to elucidate the effect.

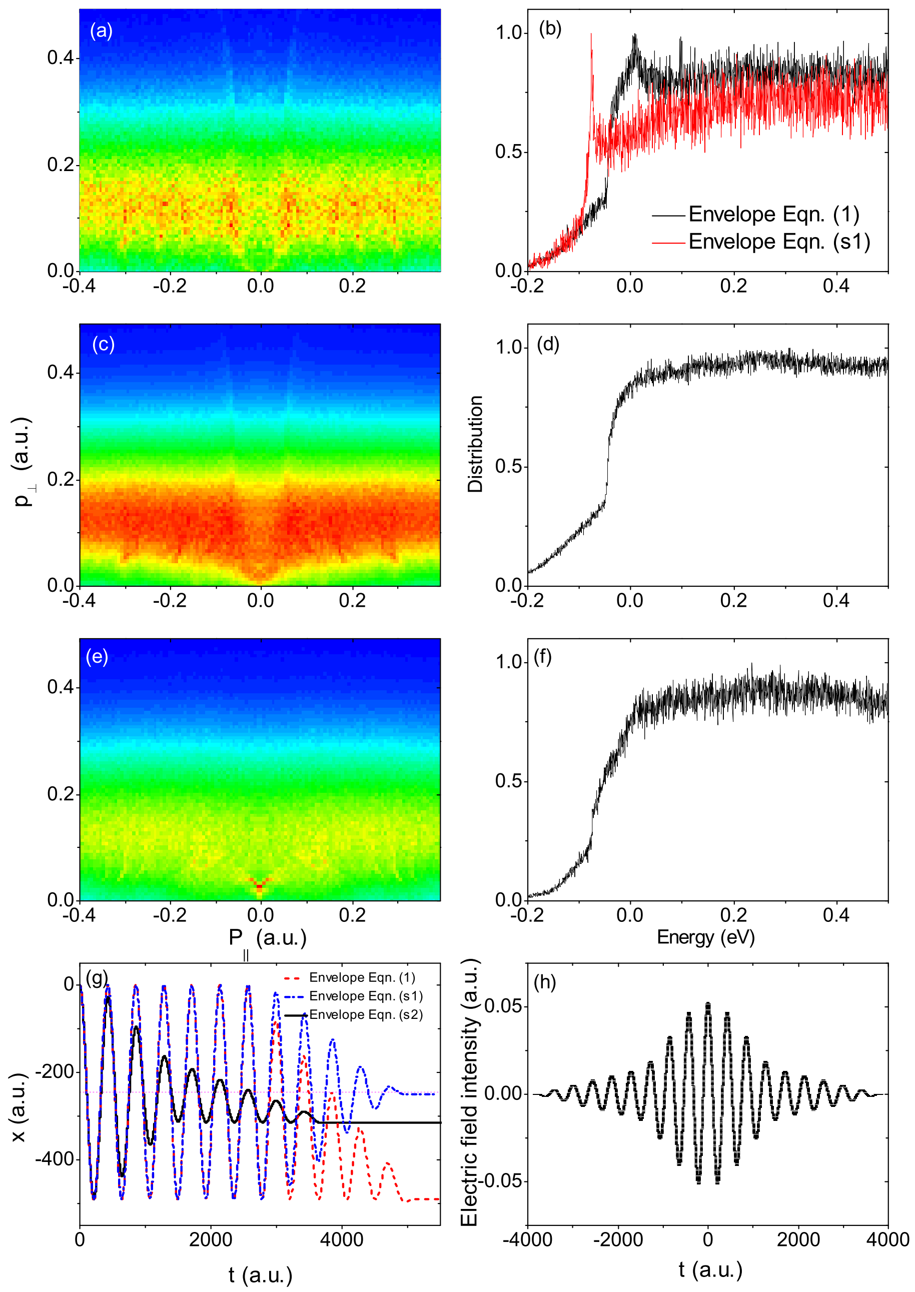

Fig. S1(Color online) (a) The momentum map of the photoelectrons originating from the first cycle of cos-squared envelope equation (s1); (b) energy distribution of the electrons originating from the first cycle of half-trapezoidal envelope as equation (1) (black curve) and cos-squared envelope as equation (s1);

(c) and (d) the momentum map and the energy distribution of the electrons originating from all cycles of full-trapezoidal 6+6+6 envelope ; (e) and (f) the momentum map and the energy distribution of the electrons originating from all cycles of hybrid envelope equation (s2). (g) the trajectory of electron released at $t = 0$ in the half-trapezoidal envelope equation (1) (red dashed curve), cos-squared envelope equation (s1) (blue dotted curve) and bell-shape envelope equation (s2) (black curve). The magenta line represents the position $x = -\frac{\varepsilon_0}{\omega^2}$. (h) the electric field of hybrid bell-shape envelope in equation (s2).

1. Ivanov, I. A. *et al.* Displacement effect in strong-field atomic ionization by an XUV pulse. *Phys. Rev. A* **90**, 043401 (2014).

2. Brabec, T., Ivanov, M. Y. & Corkum, P. B. Coulomb focusing in intense field atomic processes. *Phys. Rev. A* **54**, R2551 (1996).

3. Quan, W. *et al.* Classical Aspects in Above-Threshold Ionization with a Mid-infrared Strong Laser Field. *Phys. Rev. Lett.* **103**, 093001 (2009).

4. Liu, C. & Hatsagortsyan, K. Z. Origin of unexpected low energy structure in photoelectron spectra induced by midinfrared strong laser fields. *Phys. Rev. Lett.* **105**, 113003 (2010).
